# Prevention of Cerebrospinal Fluid Leakage in the Anterior Transpetrosal Approach †

**DOI:** 10.3390/jcm13061718

**Published:** 2024-03-16

**Authors:** Shunsuke Shibao, Kazunari Yoshida, Ryota Sasao, Masaaki Nishimoto

**Affiliations:** 1 Department of Neurosurgery, Ashikaga Red Cross Hospital, 284-1 Yobe-cho, Ashikaga 326-0843, Tochigi, Japan; sasao.sasao.sasao@gmail.com (R.S.); mnishimoto1212@outlook.jp (M.N.); 2 Department of Neurosurgery, Keio University School of Medicine, 35 Shinano-machi, Shinjuku-ku, Tokyo 160-8582, Japan; kazrmky@keio.jp

**Keywords:** anterior transpetrosal approach, cerebrospinal fluid leakage, methods for prevention, 3D simulation

## Abstract

**Background:** The anterior transpetrosal approach (ATPA) is effective for reaching petroclival lesions, and it allows for complications such as impaired venous return and neuropathy to be resolved. However, there is still room for improvement regarding cerebrospinal fluid (CSF) leakage. Here, we aim to focus on describing specific preoperative, intraoperative, and postoperative countermeasures for preventing CSF leakage when using the ATPA. **Methods:** Eleven patients treated using the ATPA, who were treated at our hospital from June 2019 to February 2023, were included in this descriptive study. Preoperatively, we performed a 3D simulation of the opened air cells. Then, we classified patterns of dural closure into three types based on intradural manipulation and whether it involved opened air cells or not. Intraoperatively, we performed a dural closure that included the use of more-watertight sutures (DuraGen^®^) and an endoscope. Furthermore, temporal bone air cell volume measurements were performed to confirm the correlation between the volume and factors related to CSF leakage. **Results:** No postoperative CSF leakage was observed in any patient. The temporal bone air cell volumes significantly corelated with the air cells of the petrous apex, the high-risk tract in the petrous apex, and postoperative fluid collection in mastoid air cells. **Conclusions:** We have described countermeasures for preventing CSF leakage when using the ATPA. Preoperative simulations and the use of multiple-layered dural reconstructions with endoscopes could be considered more reliable methods for preventing CSF leakage when using the ATPA.

## 1. Introduction

The anterior transpetrosal approach (ATPA) is an effective way to reach lesions such as meningioma, schwannoma, and epidermoids in the petroclival region [1,2]. Complications such as venous injury and cranial nerve disturbance caused by this approach have been reduced [3]. Tomio et al. reported changes in the frequency of complications in regard to the ATPA in the 1990s, 2000s, and 2010s, with surgical death (0%, 0.8%, and 0%), temporal lobe contusion (4.9%, 2.7%, and 2.3%), otitis media (4.9%, 6.3%, and 5.7%), wound infection (0%, 0.8%, and 1.1%), facial nerve palsy (5%, 8.9%, and 3.5%), and hearing impairments (3.3%, 5.4%, and 2.4%) accounting for less than 10% [4]. However, the number of cerebrospinal fluid (CSF) leakage complications has not been reduced, and the frequency of these cases has remained above 10% for more than 33 years (14.8%, 11.6%, and 14.9%) [4]. The frequency of CSF leakage has not changed due to the method of closure for opened air cells, which is not simple [4].

In the literature regarding skull base surgery, postoperative CSF leakage has been reported to be at a rate of up to 15%. Tamura et al. reported a 14.5% CSF leakage rate and a risk factor for CSF leakage after ATPA [5]. They classified the tracts of pneumatization in the temporal bone, in detail, as follows: the typical tract, the direct tract that directly communicates with the antrum, and the unusual tract, which directly communicates with the attic, tympanic cavity, and Eustachian tube [5]. A direct and unusual pattern of air cell tracts was identified as a risk factor for CSF leakages [5]. In another article on CSF leakage after the ATPA was employed, Adachi et al. analyzed the effect of spinal drainage (SD) on postoperative CSF leakage prevention, and they concluded that removing the SD immediately after surgery is recommended to avoid complications and improve patient outcomes [6].

The methods for preventing postoperative CSF leakages when using the anterior transpetrosal approach are controversial and vary between institutions. Here, we focus on our methods for CSF leakage prevention, using the ATPA method, based on our experiences; we propose a dural closure protocol that depends on lesions or ATPA modifications.

## 2. Methods

This study included eleven ATPA cases treated at our hospital over a four-year period, from June 2019 to February 2023. The diseases treated were petroclival meningioma, chondrosarcoma, cavernous sinus meningioma, petroclival epidermoid, and Meckel’s cave metastatic brain tumor. These cases were examined for the occurrence of CSF leakage using the following methods. 

Our institutional review board approved this retrospective study. Patient consent was not required as this study did not involve any interventions on the patients.

### 2.1. Preoperative Thin Slice CT (Figure 1)

Preoperatively, the presence of air cells in the temporal bone squamous part and petrous apex, and the thinning of the tympanic tegmen, were confirmed through thin-slice CT. When the thin-slice CT images revealed air cells in the squamous part or petrous apex, the case was classified as either a typical tract, a direct tract, or an unusual tract based on the classifications in Tamura et al. [5]. The typical tract passes the antrum via the mastoid air cell, whereas the direct tract passes the antrum without the mastoid air cell. The unusual tract passes around the tympanic cavity, without the antrum and mastoid air cells. The unusual tract includes the posterosuperior cell tract, the hypotympanic tract, the peritubal tract, and the squamous tympanic tract.

**Figure 1:**
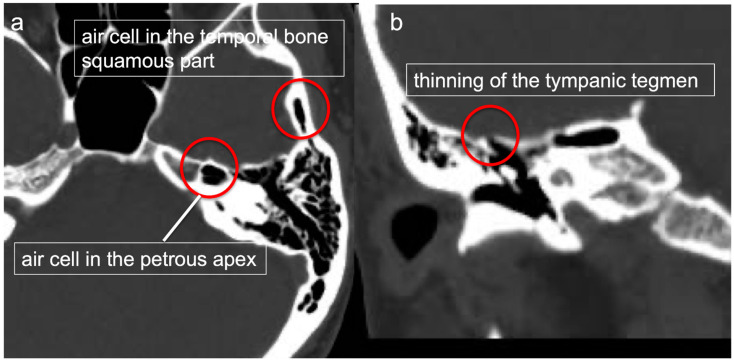
Preoperative thin-slice CT. The presence of air cells in the temporal bone squamous part and petrous apex (**a**) and thinning of the tympanic tegmen (**b**) were confirmed via thin-slice CT.

### 2.2. Preoperative 3D Simulation and Volumetric Analysis of Temporal Bone Air Cells (Figure 2)

To understand the spatial structure of the air cells around the operative field, 3D simulation was performed using Synapse Vincent^®^, a 3D simulation software product (version5.2, Fujifilm, Tokyo, Japan). A white 3D skull was created automatically by using a Synapse Vincent^®^ 3D template. Air cells of the temporal bone were semi-automatically extracted in 3D using the region-growing function and colored purple. Then, the volume of the segmented temporal bone air cells was calculated using the measurement function of Synapse Vincent^®^. 

For preoperative 3D simulation, we superimposed these elements and simulated actual ATPA procedures, including temporal craniotomy, extradural dissection of the middle cranial fossa, and drilling of the petrous apex. We then confirmed where the purple air cells would open during these procedures.

Furthermore, correlations between the temporal bone air cell volumes and factors that may be associated with postoperative CSF leakage were evaluated to elucidate the clinical significance of the temporal bone air cell volumes. Factors that may be associated with postoperative CSF leakage included the air cells of the petrous apex, tract patterns of pneumatization in the temporal bone, thinning of the tympanic tegmen, and postoperative fluid collection in the mastoid air cells or antrum detected via CT scan one day postoperatively. For tract patterns of pneumatization in the temporal bone, we defined direct tracts as high-risk tracts in the petrous apex and unusual tracts as high-risk tracts in the squamous part of the temporal bone.

**Figure 2:**
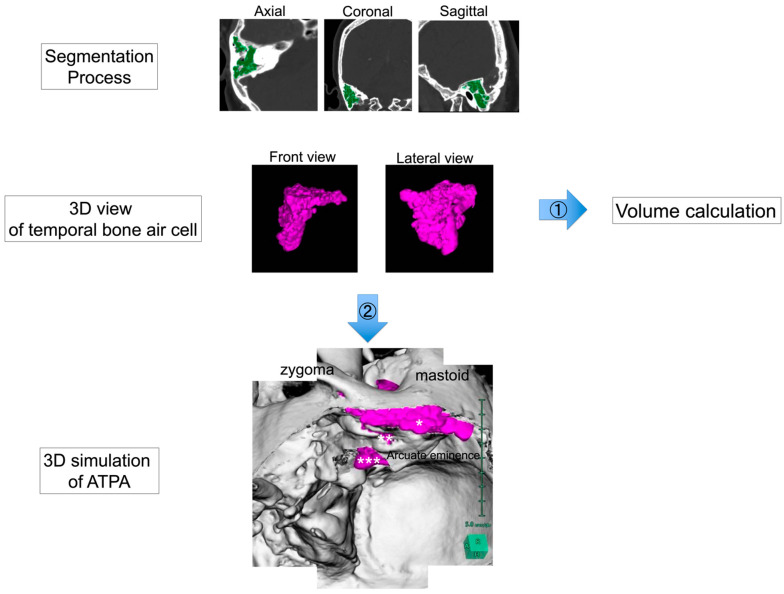
Preoperative 3D simulation and volumetric analysis of temporal bone air cells. Air cells of the temporal bone were semi-automatically extracted in 3D using the region-growing function and colored purple. The volume of the segmented temporal bone air cells was calculated using the measurement function of Synapse Vincent^®^ (Flow 1). Preoperative 3D simulation was performed to understand the three-dimensional structure of the air cells (Flow 2). Right anterior transpetrosal approach is shown. The bone, which was created automatically using a Synapse Vincent^®^ 3D template, was colored white, and the air cells were colored purple to check if they opened when craniotomy or drilling was simulated. In particular, it was confirmed whether the air cells of the craniotomy margin (*), tympanic tegmen (**), and petrous apex (***) were opened.

### 2.3. Intraoperative Precautions (Figure 3)

To prevent otitis media exudation, the intraoperatively opened air cells were temporarily filled with bone wax to prevent intraoperative water from entering them.

The actual location of the opened air cells was determined intraoperatively. Blind spots in the surgical field were checked using an endoscope.

**Figure 3:**
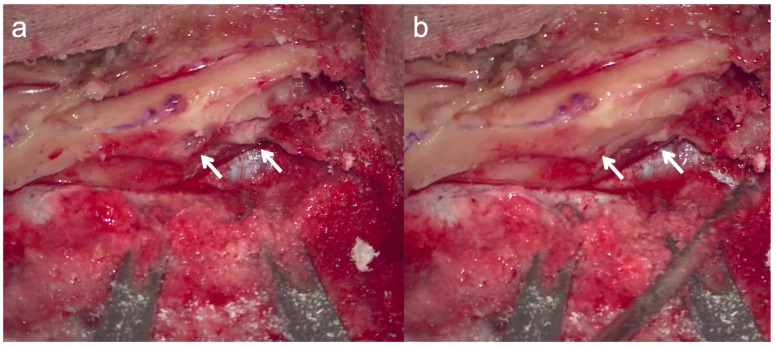
Temporary closure of opened air cells. To prevent otitis media exudation, the intraoperatively opened air cells (**a**) were temporarily filled with bone wax to prevent intraoperative water from entering the air cells. Arrows indicate areas where bone wax was applied to the opened air cells (**b**).

### 2.4. Dural Closure (Figure 4)

In the standard ATPA procedure, the dura of the middle and posterior cranial fossa was opened above and below the superior petrosal sinus. These regions were closed using the temporal fascial flap, which was harvested during skin incision. The temporal fascial flap was incised freely according to the size of the dural defect, and the dural defect was sutured and patched as much as possible. Additionally, DuraGen^®^ (Integra, Princeton, NJ, USA) was attached and used as a scaffold for dural neogenesis. Moreover, the bony defect at the petrous apex was filled with fat, and fibrin glue was applied. 

**Figure 4:**
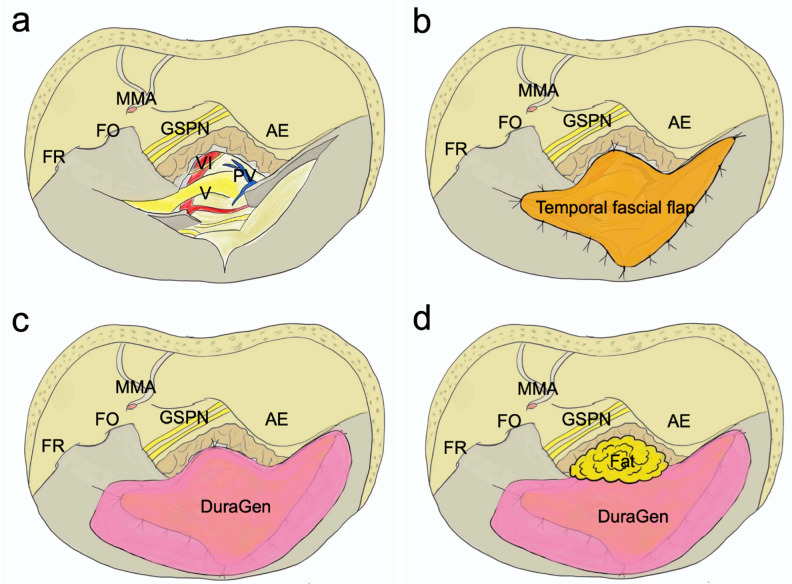
Dural closure in standard ATPA. The dura of the middle and posterior cranial fossa was opened above and below the superior petrosal sinus (**a**). These regions were closed using the temporal fascial flap, which was harvested during skin incision. The temporal fascial flap was incised freely according to the size of the dural defect, and the dural defect was sutured and patched as much as possible (**b**). In addition, DuraGen^®^ was attached and used as a scaffold for dural neogenesis (**c**). The bony defect at the petrous apex was filled with fat (**d**). AE, arcuate eminence; FO, foramen ovale; FR, foramen rotundum; GSPN, greater superficial petrosal nerve; MMA, middle meningeal artery; PV, petrosal vein; V, trigeminal verve; VI, abducens nerve.

### 2.5. Classification of Dural Closure Patterns Based on Variations of the ATPA

The pattern of dural closure was divided into three types according to intradural manipulation and whether there were opened air cells or not: cases without intradural manipulation (e.g., epidural tumor) (Type 1), cases with intradural manipulation and no opening of the petrous apex air cell (Type2), and cases with intradural manipulation and opening of the petrous apex air cell (Type3).

For each of these cases, the method of dural closure is shown.

Type 1: Cases without intradural manipulation.

Because the dura is not opened, dural closure is not necessary. The dead space where the bone was removed should be filled with Surgicel^®^ (Johnson & Johnson, New Brunswick, NJ, USA) or fat.

Type 2: Cases with intradural manipulation and no opening of petrous apex air cells.

Since the dura is open, it should be closed by patching the temporalis fascia flap and suturing it as much as possible. After removing the petrous apex, the dead space is filled with Surgicel^®^ or fat.

Type 3: Cases with intradural manipulation and opening of petrous apex air cells.

This type is most prone to CSF leakage. Since the dura mater is open, it should be closed using a temporal fascia flap, patched, and sutured as much as possible. The opened air cells at the petrous apex resection need to be densely filled with a small piece of fat (see next section for details). In addition, the dead space remaining after removing the petrous apex should be filled with fat.

### 2.6. Closure of an Opened Air Cell Cavity (Figure 5)

It is important to close the opened air cells without failure. First, autologous tissue such as abdominal fat or temporalis muscle should be used as the closure material. If the entrance of the opened air cells is small, the gap should be enlarged by drilling, allowing the autologous tissue to adhere properly. Furthermore, an endoscope was used to check the opened air cells for any missed filling and to inspect the blind spots in the surgical field (Figure 6).

**Figure 5:**
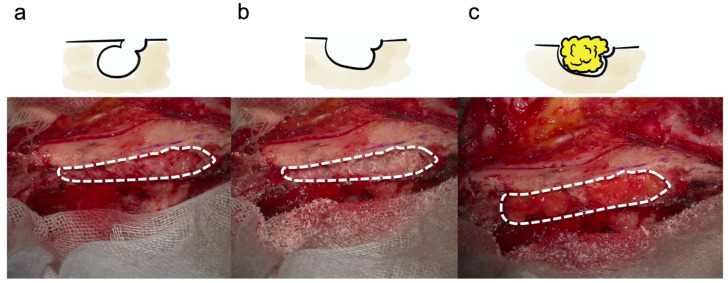
Closure of an opened air cell cavity. The air cells of the cranial margin are open (**a**). The air cells of the cranial margin have been enlarged by drilling (**b**). Filling the opened air cells of the cranial rim with fat (**c**). Dashed lines indicate an opened air cell cavity.

**Figure 6:**
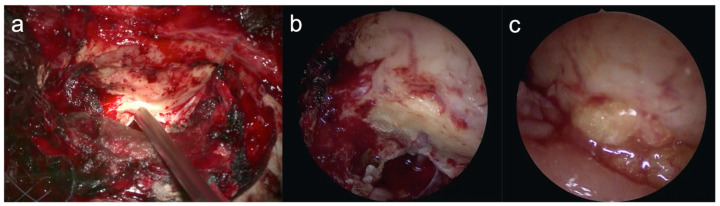
Endoscopic confirmation of air cells in a blind spot. Confirmation of the opened air cells in the blind spot at the petrous apex using an endoscope (**a**,**b**). Endoscopic confirmation that the air cells in the blind spot of the petrous apex were filled with fat (**c**).

### 2.7. Postoperative Spinal Drain (SD)

If the petrous apex air cells are opened intraoperatively or are expected to open before surgery, SD should be placed. The volume of spinal fluid drainage should be 50–200 mL, and the SD should be kept in place for 4–5 days, including the day of surgery.

### 2.8. Statistics Analysis

The normality of the data was assessed using the Shapiro–Wilk test. Variables with a normal distribution were compared using Student’s *t*-test, while those without normal distribution were compared using the Mann–Whitney U test. Differences were considered significant at *p* < 0.05.

## 3. Results

The characteristics of the patients are shown in Table S1. Their ages ranged from 35 to 76 years, with an average age of 54 years. Three patients were male, and eight were female. The pathologies included eight cases of meningioma, one case of an epidermoid, one case of chondrosarcoma, and one case of a metastatic brain tumor. Preoperative thin-slice CT showed air cells at the petrous apex in five cases and air cells at the temporal bone squamous part in all cases. The tract patterns of the five cases with air cells at the petrous apex were of the direct type in one case and of the unusual type (peritubal type 2, posterosuperior cell type 1, and hypotympanic type 1) in four cases. The tract patterns of the 11 cases with air cells at the temporal bone squamous part were typical type in seven cases and unusual (the squamous tympanic type) in four cases. Thinning of the tympanic region was observed in four cases. The patterns of dural closure were Type 1 in one case, Type 2 in five cases, and Type 3 in five cases. SD was placed in five cases. No postoperative CSF leakage occurred when using our prevention strategy.

### Volumetric Analysis of Temporal Bone Air Cells

The mean volume of temporal bone air cells was 12.4 mL (5.0–20.8 mL). 

**Figure 7:**
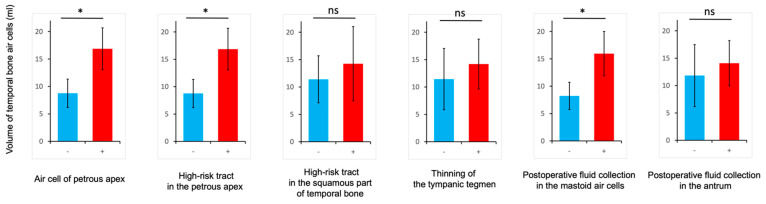
Clinical significance of the temporal bone air cell volumes. Correlations between the temporal bone air cell volumes and the air cell of the petrous apex, high-risk tract tracts in the petrous apex and the squamous part of temporal bone, thinning of the tympanic tegmen, and postoperative fluid collection in the mastoid air cells or antrum. * *p* < 0.05. ns, not significant.

As for correlations between the temporal bone air cell volumes and factors that may be associated with postoperative CSF leakage, cases with air cells of the petrous apex, a high-risk tract in the petrous apex, and postoperative fluid collection in the mastoid air cells showed significantly larger temporal bone air cell volumes compared to those without these factors (Figure 7).

## 4. Discussion

### 4.1. CSF Leakage in Skull Base Surgery

The frequency of CSF leakage in skull base surgery has been reported to be 4–15% [5,7,8,9,10,11]. Perry et al. reported a significant quantity of data about CSF leakage in skull base surgery [12]. They analyzed 2918 patients suffering from skull base issues including meningioma, schwannoma, pituitary adenoma, and trigeminal neuralgia (TN). They found CSF leakage requiring rehospitalization and retreatment in 2.9% of patients and identified schwannoma, TN, chronic obstructive pulmonary disease, and increased operative time as risk factors for CSF leakage [12]. Other risk factors for CSF leakage in skull base surgery include pneumatization of the petrous apex [11], the development of air cells [7], recurrent surgery [8], thinning of the tympanic tegmen [10], and unique air cell pathways [5]. Risk factors for CSF leakage when using ATPA include pneumatized petrous bone and direct squamous tracts [5].

Traditional methods for preventing CSF leakage in skull base surgery have been reported; however, we have described specific methods that include preoperative simulation as a more reliable way to prevent CSF leakage when using the ATPA. Furthermore, we were able to prevent CSF leakage even in patients with high-risk factors for CSF leakage in our case series.

### 4.2. CSF Leakage in ATPA

While CSF leakage is considered a low-risk complication in the ATPA compared to its occurrence in the combined transpetrosal approach, it is an issue that surgeons should be aware of. Studies by Fukaya et al. and Van Gompel et al. reported a CSF leak incidence of about 4% in the ATPA [13,14]. In the series by Volovici et al., the risk of CSF leakage when using the ATPA was reported to be 28% [15].

CSF leakage into the air cells can cause exudative otitis media and hearing impairment, and to prevent this, as described in this paper, the opened air cells should be temporarily closed intraoperatively with bone wax and then closed with autologous tissue such as fat or muscle fragments during wound closure. Regarding closure of the petrous apex, we previously used an autologous fat graft and fibrin glue to pack the opened air cells and cover the temporalis base with a fascial flap of the temporalis muscle to prevent CSF leakage. However, the incidence of CSF leakage has remained at about 14% since the 1980s, and about 17% of cases have required revision surgery [4]. The methods employed in our case series represent a partial solution to this problem. The literature on CSF leak prevention methodology covers the preoperative identification of the air cells in temporal bone, covering the tympanic membrane with bone wax and pericranial fragments, the complete filling of opened air cells with appropriately sized fat grafts, visualization of the open air cells with an endoscope, pulling the dura as close as possible, filling the space between the dead space and the dura with fat as much as possible, and fibrin gluing [16,17]. In this paper, we show that we have succeeded in preventing CSF leakage by incorporating the mastoid air cell 3D simulation and multiple layer reconstruction using DuraGen^®^ in addition to previous proposed methods. Table S2 compares the incidence of CSF leakage and methods of CSF leakage prevention when using the ATPA between our case series and the previous literature on ATPA outcomes [4,5,6,14,15,16,18]. The incidence of CSF leakage does not show a trend of gradual improvement with age, although it is important to clarify the definition of CSF leakage. It is likely that each institution adopts measures to prevent CSF leakage based on their own cases, and the methods used for prevention can only be used as a reference. However, our policy is to do everything we can to prevent CSF leaks because, in high-risk cases of CSF leakage, it is important to ensure the closure of opened air cells. Our study is unique in that we used new methods of CSF leak prevention not previously reported, such as preoperative 3D simulation, the use of DuraGen^®^, and endoscopy.

### 4.3. 3D Simulation to Prevent CSF Leakage When Using the ATPA

Preoperative 3D simulation has been reported to be useful in preoperative skull base surgeries. Recently, preoperative simulation using a 3D printer has also been reported [19]. To the best of our knowledge, there have been no reports of the use of preoperative imaging simulation specifically for CSF leakage prevention. We used 3D simulation to predict the presence or absence of the intraoperative opening of air cells. While it is difficult to predict how they will be opened during an actual craniotomy or the removal of the petrous apex in 2D, by simulating these procedures in 3D before surgery, it is possible to intuitively understand exactly how the air cells will be opened. Although 3D printing was not used in this study, 3D simulation alone is considered sufficient for confirming the presence or absence of the intraoperative opening of air cells, considering time and cost. This method proves useful for avoiding missing the intraoperative closure of open air cells.

### 4.4. Volumetric Analysis of Temporal Bone Air Cells

To the best of our knowledge, while some reports have measured temporal bone air cell volumes, none have shown a relationship between temporal bone air cell volumes and CSF-leakage-related factors [20,21,22,23]. This is the first study to demonstrate the relationship between air cell volumes and factors related to CSF leakage. In the measurement of temporal bone air cell volume, there was a significant correlation between temporal bone air cell volumes and CSF-leakage-related factors such as air cells of the petrous apex, a high-risk tract in the petrous apex, and postoperative fluid collection in the mastoid air cells. This is because a greater number of air cells corresponds to a more dangerous pattern of pneumatization in the temporal bone, resulting in a larger volume of temporal bone air cells. As a consequence, postoperative fluid collection in the mastoid air cells also increases. These findings suggest that measurements of temporal bone air cell volume may be instrumental in more reliably reducing the risk of CSF leakage. In addition, by measuring the volume of the air cells to be opened, it becomes possible to harvest autologous tissues such as fat and muscle fragments that fit more appropriately.

### 4.5. Dural Closure in ATPA

As for dural closure methods, achieving a complete watertight suture of the dura mater is not possible when using the ATPA. Among the methods reported, Adachi et al. suggested that filling the opened air cells with abdominal fat may be effective in the management of high-flow CSF leakage. Additionally, the placement of a pericranial flap in the middle cranial fossa, cut from the posterior fossa dural defect, may be effective in the management of dural defects that exceed 1 cm^2^ in area [6]. However, achieving an as-close-to-watertight closure as possible is important, especially in case of high-flow CSF leakage. We sutured the areas that could be sutured as much as possible and performed multiple-layer reconstruction using autologous tissue such as fascia and fat, in addition to utilizing DuraGen^®^. DuraGen^®^ is an absorbable artificial dura mater made of bovine-Achilles-tendon-derived collagen that is used for dural defects. It is a spongy sheet composed of a porous collagen matrix that serves as a scaffold allowing fibroblasts to produce and deposit new collagen [17]. We used DuraGen^®^ as a scaffold for dural neoplasia by supplementing areas where watertight sutures are not possible. The application of this multiple-layer dural reconstruction method using DuraGen^®^ to the ATPA could be considered a more reliable method for preventing CSF leakage.

### 4.6. Fat Packing in Air Cells in ATPA

Air cells at the petrous apex and at the temporal bone squamous part connect to the tympanic cavity through the mastoid antrum. If these air cells remain open postoperatively, they can cause postoperative CSF leakage. These air cell cavities should be closed by filling them with autologous tissue, such as fat or muscle fragments, during closure. However, if there is a small gap, there is a risk of failing to fill the corresponding area. To prevent this, we endoscopically identified small gaps and enlarged them with a drill to ensure that an area could be filled with fat. Additionally, smaller pieces of fat were used to fill the deeper sites of the opened air cells to match the size of the air cell cavity in the front. Adachi et al. reported that the use of small abdominal fat is beneficial because its plasticity allows it to be inserted into an opened air cell without interfering with the visibility of the deep operative field [6].

Our previous method of preventing CSF leakage was reported by our colleagues Tomio et al. and Tamura et al. In their studies, 3D simulation, watertight sutures, DuraGen^®^, and endoscopy were not used. The reported frequency of CSF leakage was between 14.5 and 14.9% [4,5]. In summary, the novelty of our current dural closure method lies in its integration of the use of 3D simulation, more watertight sutures, DuraGen^®^, and an endoscope. 

### 4.7. Spinal Drain (SD)

The effectiveness of SD placement after skull base surgery has been reported in regard to craniotomy and endoscopic endonasal surgery [24,25,26,27,28,29]. SD placement is considered necessary to prevent postoperative CSF leakage, especially in cases of high CSF pressure and large dural defects [29,30]. In a meta-analysis evaluating the efficacy of postoperative SD placement in preventing CSF leakage in patients undergoing endoscopic endonasal skull base surgery, the incidences of CSF leakage were 8.2% and 21.2% in the groups with and without SD implantation, respectively [29]. Adachi et al. reported that removing the SD immediately after surgery is recommended to avoid complications and improve patient outcomes, leading to earlier ambulation and shorter hospital stays [6]. They concluded that preventive postoperative SD placement is unnecessary. We do not routinely use SDs. However, given that cases with opened petrous apex air cells pose a high risk of CSF leakage, we attempted to insert an SD only in these cases to prevent CSF leakage. 

### 4.8. Patterns of Dural Closure Classified Based on the Variation of ATPA

The ATPA requires different procedures depending on the localization of a lesion. For example, since epidural lesions without intradural extension, such as chordoma and chondrosarcoma, may not require a dural incision, the risk of CSF leakage is low even with a simple closure procedure. On the other hand, removal of intradural lesions such as meningioma, schwannoma, and epidermoid involves a dural incision, which results in a dural defect and the need for dural reconstruction. Furthermore, in the ATPA, pneumatized petrous bone has been reported to be a risk factor for CSF leakage, as mentioned above, and requires meticulous dural reconstruction. For the aforementioned reasons, we have classified the patterns of dural closure in the ATPA into three categories: cases without intradural manipulation (Type 1), cases with intradural manipulation and no opening of petrous apex air cells (Type 2), and cases with intradural manipulation and opening of petrous apex air cells (Type 3). While meticulous reconstruction of the dura mater may not be necessary in Type 1, it is essential in Types 2 and 3. Since Type 3, in particular, is poses a high risk of CSF leakage [30], it requires meticulous fat filling of the opened air cells at the petrous apex, in addition to multiple-layer dural reconstruction. 

### 4.9. Limitations 

One limitation of this study is the limited number of cases investigated. The ATPA is not a widely performed procedure, and general neurosurgeons have limited experience with it. While it is not possible to conclusively assert that this method has prevented 100% of CSF leakage due to the small number of cases, it is necessary to verify this by increasing the number of cases analyzed in the future. However, the method of preventing CSF leakage was derived from the accumulated experience with ATPA of one of the co-authors, K.Y. [4], and is considered to be a reliable method.

## 5. Conclusions

We have described countermeasures for preventing CSF leakage when using the ATPA based on our experience. Preoperative simulation and multiple-layered dural reconstruction using endoscope are considered novel methods for preventing CSF leakage when using the ATPA. The effectiveness of this procedure should be evaluated in more cases in the future.

## Data Availability

The original contributions presented in the study are included in the article/Supplementary Material, further in-quiries can be directed to the corresponding author.

## Supplementary Materials

The following supporting information can be downloaded at: https://www.mdpi.com/article/10.3390/jcm13061718/s1, Table S1. Patients characteristics in ATPA; Table S2. Comparison of the incidence of CSF leakage and methods of CSF leakage prevention in ATPA among previous literatures.

## Abbreviations

ATPA: anterior transpetrosal approach; CSF: cerebrospinal fluid; SD: spinal drain.

## References

[1] Kawase T., Shiobara R., Toya S. (1991). Anterior transpetrosal-transtentorial approach for sphenopetroclival meningiomas: Surgical method and results in 10 patients. Neurosurgery.

[2] Kawase T., Toya S., Shiobara R., Mine T. (1985). Transpetrosal approach for aneurysms of the lower basilar artery. J. Neurosurg..

[3] Shibao S., Toda M., Orii M., Fujiwara H., Yoshida K. (2016). Various patterns of the middle cerebral vein and preservation of venous drainage during the anterior transpetrosal approach. J. Neurosurg..

[4] Tomio R., Horiguchi T., Borghei-Razavi H., Tamura R., Yoshida K., Kawase T. (2021). Anterior transpetrosal approach: Experiences in 274 cases over 33 years. Technical variations, operated patients, and approach-related complications. J. Neurosurg..

[5] Tamura R., Tomio R., Mohammad F., Toda M., Yoshida K. (2018). Analysis of various tracts of mastoid air cells related to CSF leak after the anterior transpetrosal approach. J. Neurosurg..

[6] Adachi K., Hasegawa M., Hirose Y. (2023). Cerebrospinal fluid leakage prevention using the anterior transpetrosal approach with versus without postoperative spinal drainage: An institutional cohort study. Neurosurg. Rev..

[7] Fishman A.J., Marrinan M.S., Golfinos J.G., Cohen N.L., Roland J.T. (2004). Prevention and management of cerebrospinal fluid leak following vestibular schwannoma surgery. Laryngoscope.

[8] Nanda A., Javalkar V., Banerjee A.D. (2011). Petroclival meningiomas: Study on outcomes, complications and recurrence rates. J. Neurosurg..

[9] Nutik S.L., Korol H.W. (1995). Cerebrospinal fluid leak after acoustic neuroma surgery. Surg. Neurol..

[10] Stevens S.M., Rizk H.G., McIlwain W.R., Lambert P.R., Meyer T.A. (2016). Association between Lateral Skull Base Thickness and Surgical Outcomes in Spontaneous CSF Otorrhea. Otolaryngol. Head Neck Surg..

[11] Yamakami I., Uchino Y., Kobayashi E., Yamaura A. (2003). Computed tomography evaluation of air cells in the petrous bone--relationship with postoperative cerebrospinal fluid rhinorrhea. Neurol. Med. Chir..

[12] Perry A., Kerezoudis P., Graffeo C.S., Carlstrom L.P., Peris-Celda M., Meyer F.B., Bydon M., Link M.J. (2019). Little Insights from Big Data: Cerebrospinal Fluid Leak After Skull Base Surgery and the Limitations of Database Research. World Neurosurg..

[13] Fukaya R., Yoshida K., Ohira T., Kawase T. (2010). Trigeminal schwannomas: Experience with 57 cases and a review of the literature. Neurosurg. Rev..

[14] Van Gompel J.J., Alikhani P., Youssef A.S., Loveren H.R., Boyev K.P., Agazzi S. (2015). Anterior Petrosectomy: Consecutive Series of 46 Patients with Attention to Approach-Related Complications. J. Neurol. Surg. B Skull Base.

[15] Volovici V., Dammers R., Dirven C.M.F., Delwel E.J. (2020). Conquering the Rock-A Retrospective Single-Center Experience of the Transapical Petrosal Transtentorial (Kawase) Approach: Operative Technique and Impact on Cranial Nerve Function. J. Neurol. Surg. B Skull Base.

[16] Giammattei L., Passeri T., Abbritti R., Lieber S., Matano F., Le Van T., Okano A., Fava A., di Russo P., Froelich S. (2023). Surgical morbidity of the extradural anterior petrosal approach: The Lariboisiere experience. J. Neurosurg..

[17] Nagata Y., Takeuchi K., Sasaki H., Mizuno A., Harada H., Tanahashi K., Araki Y., Saito R. (2022). Modified Shoelace Dural Closure with Collagen Matrix in Extended Transsphenoidal Surgery. Neurol. Med. Chir..

[18] Cheng Y., Song Y., Yang W., Wang L., Li X., Bai J., Xiao X. (2023). The Evolution of Anterior Transpetrosal Approach for the Treatment of Petroclival Meningiomas: A Single-Center 128-Case Experience. World Neurosurg..

[19] Neves C.A., Tran E.D., Kessler I.M., Blevins N.H. (2021). Fully automated preoperative segmentation of temporal bone structures from clinical CT scans. Sci. Rep..

[20] Adisen M.Z., Aydogdu M. (2022). Comparison of mastoid air cell volume in patients with or without a pneumatized articular tubercle. Imaging Sci. Dent..

[21] Aladeyelu O.S., Olojede S.O., Lawal S.K., Matshipi M.N., Sibiya A.L., Rennie C.O., Mbatha W.E. (2023). Three-dimensional volumetric analyses of temporal bone pneumatization from early childhood to early adulthood in a South African population. Folia Morphol..

[22] Nishida E., Sakaida H., Kitano M., Takeuchi K. (2024). Quantification of Mastoid Air Cells and Opacification of the Middle Ear in Primary Ciliary Dyskinesia. Otol. Neurotol..

[23] Oura K., Ikeda N., Yoon Y., Kato T., Morishita J. (2022). Potential for personal identification using the volume of the mastoid air cells extracted from postmortem computed tomographic images. Leg. Med..

[24] Bien A.G., Bowdino B., Moore G., Leibrock L. (2007). Utilization of preoperative cerebrospinal fluid drain in skull base surgery. Skull Base.

[25] Dalle Ore C.L., Magill S.T., Rodriguez Rubio R., Shahin M.N., Aghi M.K., Theodosopoulos P.V., Villanueva-Meyer J.E., Kersten R.C., Idowu O.O., Vagefi M.R. (2020). Hyperostosing sphenoid wing meningiomas: Surgical outcomes and strategy for bone resection and multidisciplinary orbital reconstruction. J. Neurosurg..

[26] Forbes J.A., Ordonez-Rubiano E.G., Tomasiewicz H.C., Banu M.A., Younus I., Dobri G.A., Phillips C.D., Kacker A., Cisse B., Anand V.K. (2018). Endonasal endoscopic transsphenoidal resection of intrinsic third ventricular craniopharyngioma: Surgical results. J. Neurosurg..

[27] Hussein M., Abdellatif M. (2019). Continuous Lumbar Drainage for the Prevention and Management of Perioperative Cerebrospinal Fluid Leakage. Asian J. Neurosurg..

[28] Tan J., Song R., Huan R., Huang N., Chen J. (2020). Intraoperative lumbar drainage can prevent cerebrospinal fluid leakage during transsphenoidal surgery for pituitary adenomas: A systematic review and meta-analysis. BMC Neurol..

[29] Zwagerman N.T., Wang E.W., Shin S.S., Chang Y.F., Fernandez-Miranda J.C., Snyderman C.H., Gardner P.A. (2018). Does lumbar drainage reduce postoperative cerebrospinal fluid leak after endoscopic endonasal skull base surgery? A prospective, randomized controlled trial. J. Neurosurg..

[30] Kreatsoulas D.C., Shah V.S., Otto B.A., Carrau R.L., Prevedello D.M., Hardesty D.A. (2020). Surgical outcomes of the endonasal endoscopic approach within a standardized management protocol for repair of spontaneous cerebrospinal fluid rhinorrhea. J. Neurosurg..

