# Supplementary material for: Prevention of Cerebrospinal Fluid Leakage in the Anterior Transpetrosal Approach
Source: J Clin Med. 2024 Mar 16;13(6):1718. doi: 10.3390/jcm13061718 (PMC10971171; doi:10.3390/jcm13061718)
Supplement: Supplementary file 1 [file jcm-13-01718-s001.zip › jcm-2759526-supplementary.pdf]

Table S1. Patients characteristics in ATPA

No	Age	Sex	Location	Pathology	Air cell of petrous apex (PA)	Tract pattern (PA)	Air cell in squamous part of temporal bone (TB)	Tract pattern (TB)	Thin tympanic tegmen	Dural closure pattern	Spinal drainage	CSF leak
1	35	M	Petrous	Chondrosarcoma	-	-	+	Typical	+	Type 1	-	-
2	45	M	Petroclival	Meningioma	-	-	+	Typical	-	Type 2	-	-
3	46	F	Cavernous sinus	Meningioma	+	Direct	+	Typical	+	Type 3	+	-
4	70	F	Meckel's cave	Metastatic tumor	+	Unusual (Peritubal)	+	Typical	-	Type 3	+	-
5	56	F	Petroclival	Meningioma	+	Unusual (Peritubal)	+	Unusual (Squamous tympanic)	-	Type 3	+	-
6	50	F	Petroclival	Meningioma	-	-	+	Typical	-	Type 2	+	-
7	68	F	Petroclival	Meningioma	-	-	+	Unusual (Squamous tympanic)	-	Type 2	-	-
8	53	M	Petroclival	Meningioma	+	Unusual (posterosuperior cell)	+	Typical	-	Type 3	-	-
9	41	F	Petrous	Meningioma	-	-	+	Unusual (Squamous tympanic)	+	Type 2	-	-
10	55	F	Petroclival	Meningioma	+	Unusual (Hypotympanic)	+	Unusual (Squamous tympanic)	+	Type 3	+	-
11	76	F	Petroclival	Epidermoid	-	-	+	Typical	-	Type 2	-	-

Table S2. Comparison of the incidence of CSF leakage and methods of CSF leakage prevention in ATPA among previous literatures

Author	Year	N	CSF leak(%)	Methods							
				Preoperative imaging check	Preoperative 3D simulation	Flap	Fat	Fiblin glue	DuraGen	Endoscopy	Postoperative spinal drainage
Van Gompel [27]	2015	46	4	NA	NA	NA	NA	NA	NA	NA	NA
Tamura [24]	2019	117	14.5	+	-	+	+	+	-	-	+
Volovici [28]	2020	25	28	-	-	+	+	+	-	-	+
Tomio [26]	2021	274	13.5	+	-	+	+	+	-	-	+
Adachi [1]	2023	48	0	+	-	+	+	+	-	-	-
Cheng [5]	2023	128	3.1	-	-	+	NA	NA	NA	+	+
Giammattei [10]	2023	49	0	+	-	+	+	+	-	-	-
Present series	2023	11	0	+	+	+	+	+	+	+	+

References

1. Adachi K, Hasegawa M, Hirose Y (2023) Cerebrospinal fluid leakage prevention using the anterior transpetrosal approach with versus without postoperative spinal drainage: an institutional cohort study. Neurosurg Rev 46:137
5. Cheng Y, Song Y, Yang W, Wang L, Li X, Bai J, Xiao X (2023) The Evolution of Anterior Transpetrosal Approach for the Treatment of Petroclival Meningiomas: A Single-Center 128-Case Experience. World Neurosurg
10. Giammattei L, Passeri T, Abbritti R, Lieber S, Matano F, Le Van T, Okano A, Fava A, di Russo P, Froelich S (2023) Surgical morbidity of the extradural anterior petrosal approach: the Lariboisiere experience. J Neurosurg 138:276-286
24. Tamura R, Tomio R, Mohammad F, Toda M, Yoshida K (2018) Analysis of various tracts of mastoid air cells related to CSF leak after the anterior transpetrosal approach. J Neurosurg 130:360-367
26. Tomio R, Horiguchi T, Borghei-Razavi H, Tamura R, Yoshida K, Kawase T (2021) Anterior transpetrosal approach: experiences in 274 cases over 33 years. Technical variations, operated patients, and approach-related complications. J Neurosurg:1-9
27. Van Gompel JJ, Alikhani P, Youssef AS, Loveren HR, Boyev KP, Agazzi S (2015) Anterior Petrosectomy: Consecutive Series of 46 Patients with Attention to Approach-Related Complications. J Neurol Surg B Skull Base 76:379-384
28. Volovici V, Dammers R, Dirven CMF, Delwel EJ (2020) Conquering the Rock-A Retrospective Single-Center Experience of the Transapical Petrosal Transtentorial (Kawase) Approach: Operative Technique and Impact on Cranial Nerve Function. J Neurol Surg B Skull Base 81:526-535
